# Inhaled nitric oxide prevents NSAID-induced renal impairment in pseudo-normovolaemic piglets

**DOI:** 10.1371/journal.pone.0179475

**Published:** 2017-06-28

**Authors:** Stephane Junot, Stephanie Keroak, Jerome R. E. Del Castillo, Jean-Yves Ayoub, Christian Paquet, Jeanne-Marie Bonnet-Garin, Eric Troncy

**Affiliations:** 1 VetAgro Sup - Veterinary Campus of Lyon - University of Lyon, APCSE unit, Marcy l’Etoile, France; 2 Faculty of Veterinary Medicine - University of Montreal, GREPAQ (Research group in animal pharmacology of Quebec) - Department of Veterinary Biomedicine, Saint-Hyacinthe, Québec, Canada; University of Alabama at Birmingham, UNITED STATES

## Abstract

**Objective:**

Inhaled nitric oxide (iNO) is commonly used as a treatment of pulmonary hypertension. Its action is purported to be specific to the lung, but extrapulmonary effects have been reported. The objective of this study was to evaluate if iNO could compensate the renal impairment induced by ketoprofen, a conventional non-steroidal anti-inflammatory drug (NSAID), during general anaesthesia.

**Methods:**

Under pseudo-normovolaemic condition, thirty piglets were randomly assigned into 5 equal groups and equipped for renal and systemic parameters measurements. A first experiment was carried out to validate methods and reproduce the renal effects of iNO (40 ppm) in comparison with a placebo (100% oxygen). In a second experiment, iNO was inhaled for 120 minutes right after NSAID treatment (ketoprofen 2 mg×kg^-1^ IV, and 40 ppm iNO; group KiNO) and its effects were compared to ketoprofen alone (2 mg×kg^-1^ IV; group K) and placebo (saline; group C).

**Results:**

In this model, iNO increased significantly renal blood flow measured by ultrasonic (RBF_UL_: +53.2±17.2%; p = 0.008) and by PAH clearance (RBF_PAH_:+78.6±37.6%; p = 0.004) methods, glomerular filtration rate (GFR: +72.6±32.5%; p = 0.006) and urinary output (UO: +47.4±24.2%; p = 0.01). In the second experiment, no significant temporal variation was noted for renal parameters in groups KiNO and C, whereas a significant and constant decrease was observed in the group K for RBF_UL_ (max -19.0±7.1%), GFR (max -26.6±10.4%) and UO (max -30.3±10.5%).

**Clinical significance:**

Our experiments show that iNO, released from its transport forms after its inhalation, can improve renal safety of NSAIDs. This result is promising regarding the use of NSAIDs in critical conditions, but needs to receive clinical confirmation.

## Introduction

Acute kidney injury (AKI) is defined as a rapid deterioration in renal function, it is a common complication in the perioperative settings and is associated with an increased morbidity and mortality [1]. It has been reported in 7 to 18% of hospitalized patients [2] and approximately one out of three AKI in hospitalised patients are acquired in the perioperative setting [3]. Several risk factors for developing AKI in the perioperative period have been identified among which sepsis, hypovolaemia, chronic kidney disease and diabetes mellitus [4]. Intraoperative hypotension and the use of nephrotoxic drugs such as non-steroidal anti-inflammatory drugs (NSAIDs) have also been reported as causative factors of hospital-acquired AKI [5,6]. The blockage of prostanoid synthesis by NSAIDs, through the inhibition of the cyclooxygenase (COX) enzymes activity, explains their clinical efficacy but also their renal side effects [7,8]. In particular, the use of NSAIDs in conditions of decreased renal perfusion, such as general anaesthesia, may alter compensatory mechanisms of renal hypovolaemia and participate to the development of perioperative AKI [9]. In spite of this, NSAIDs are still widely prescribed in human and veterinary medicine for the treatment of surgical pain, as a sole agent or more often as part of a balanced analgesic protocol [10]. Preferential or selective inhibition of inducible COX-2 isoform has led to improved gastro-intestinal safety but is not denuded of renal side effects [11,12].

Therefore, prevention of NSAID-induced peri-operative AKI remains difficult. A treatment allowing renal vasodilation without affecting systemic vascular resistance could be promising, particularly in conditions of hypovolaemia or hypotension.

Nitric oxide (NO) is an endogenous mediator, strongly implied in endothelial control of vascular tonicity [13]. Alike vasodilatory prostaglandins (PGs), endogenous NO plays an important role on renal vascular tonicity by dampening the vasoconstriction induced by different factors. Due to its vasodilatory properties, NO has been evaluated as a medication in human medicine, especially when given by inhalation. Inhaled NO (iNO) is commonly used for treatment of pulmonary hypertension and severe hypoxaemia [14]. Main effects of iNO are located within the lung (*e*.*g*., on oxygenation or leukocytes trapped in the pulmonary area). However, through blood-borne molecules carriage into general circulation, iNO also has extrapulmonary effects [15,16].

With regards to the therapeutic advantages of NSAIDs for surgical pain and taking in consideration the important link between NO and prostanoid pathways, we aim to evaluate if the co-administration of NO given by inhalation, due to its extrapulmonary effects, could improve the renal tolerance to NSAIDs. We propose hereby to evaluate this assumption in an experimental animal model with water restriction, a condition that would place the kidney in a vasodilatory PGs-dependent state and would promote the renal effects of NSAIDs. The objectives of this study were: 1) to reproduce the renal effects of iNO in different experimental conditions; and mostly 2) to evaluate whether iNO could compensate or prevent the decrease in renal blood flow (RBF) induced by ketoprofen, a conventional NSAID, during general anaesthesia.

## Materials and methods

The experimental protocol described thereafter received the approbation of the Ethical and Animal Use Committee of VetAgro Sup (protocol n°1180) in order to satisfy the criteria of European regulation (Directive 2010/63/EU). The experiments were performed under randomised-blinded conditions. Both experimental phases used the same anaesthetic and surgical procedures.

### Animals

Thirty piglets (16 males), 25 to 32 kg, 75 to 90 days old, were used for this study. During their 2-weeks acclimatisation, the animals were fed with standard food, and had *ad libitum* access to water. Alternate 12-hours periods of luminosity and darkness were respected. Twelve hours before the beginning of the experimental phase, solid food was withdrawn.

### Anaesthesia and instrumentation

Animals were premedicated with intramuscular administration of ketamine (Imalgene, 100 mg.mL^-1^, Merial, Lyon, France) 15.0 mg.kg^-1^ and azaperone (Stresnil, 40 mg.mL^-1^, Elanco, Neuilly sur Seine, France) 2.0 mg.kg^-1^. Twenty min later, pentobarbital (Pentobarbital sodique, 60 mg.mL^-1^, CEVA Santé Animale, Libourne, France) 10.0 mg.kg^-1^, was administered intravenously to effect *via* a catheter placed in the ear marginal vein. Maintenance of anaesthesia was provided by an intravenous constant rate infusion (CRI) of ketamine 5.0 mg.kg^-1.^h^-1^, morphine (Morphine Aguettant, 10 mg.mL^-1^, Laboratoire Aguettant, Lyon, France) 0.1 mg.kg^-1^.h^-1^, and pentobarbital 10.0 mg.kg^-1.^h^-1^ using a pump (Volumed VP5000^®^, Arcomed, Saint-Ouen l'Aumône, France). A CRI of pancuronium (Pavulon, 2 mg.mL^-1^, Organon-Teknika, Fresnes, France), a neuromuscular blocking agent, completed the balanced anaesthesia protocol: loading dose of 0.1 mg.kg^-1^ over 2 min, followed by 0.01 mg.kg^-1^.h^-1^ CRI. After induction of anaesthesia, animals were orotracheally intubated and placed under mechanical ventilation with controlled tidal volume set at 10.0 mL.kg^-1^. The inspired fraction of oxygen was 100% and respiratory rate was adjusted to obtain an end tidal CO _2_ (ETCO_2_) of 4.6–6.0 kPa (35–45 mmHg). No fluid was administered during the experiment. The total volume of fluid injected, resulting from the CRIs, was calculated as 3.5 mL.kg^-1^.h^-1^.

The animals were placed in dorsal recumbency on a heated table. The following structures were isolated and catheterised: intravenous catheters were placed in the left saphenous vein for anaesthetic drug injection and in the left cephalic vein for infusion of inulin (I3754, Sigma-Aldrich, Saint Quentin-Fallavier, France) and para-amino hippuric acid (PAH; P-aminohippuric Acid—Sodium Salt, Merck Eurolab SAS, Fontenay-sous-Bois, France). The right carotid artery was catheterised to measure systemic arterial blood pressure (SAP) and to withdraw blood for further analysis. External right jugular vein was dissected for placement of a Swan-Ganz catheter (IAG-6 6F/CH6^®^, Prodimed, Neuilly en Thelle, France) in the pulmonary artery and monitoring of pulmonary arterial blood pressure (PAP). Through a ventral midline laparotomy, both ureters were isolated and cannulated (Medical Pipe and Surgical^®^, Tygon, Saint-Gobain Performance Plastics Corporation, Charny, France) for urine collection. A 3 mm diameter ultrasonic flow probe (T206^®^, Transonic Systems, Ithaca, NY, U.S.A.) was placed around the right renal artery. Good contact was ensured with water-soluble ointment (K-Y Jelly^®^, Johnson & Johnson, Arlington, Texas, U.S.A.). The abdomen was then sutured close (PDS II^®^, Ethicon, Issy-les-Moulineaux, France).

### Study design

#### Experimental phase I: Reproduction of inhaled NO renal effects and methods validation

Twelve piglets were used in this experiment. After a 30 minute period of stabilisation, the measurements began with a 20 minute baseline period (period BASE). At T0, 100% O_2_ without (group C; n = 6) or with 40 ppm iNO (group iNO; n = 6) was administered during 20 minutes (period GAS) followed by a recovery period (REC period) of 20 minutes without iNO administration.

#### Experimental phase II: Effect of inhaled NO during ketoprofen administration

Eighteen piglets were used for this experiment. After a 30 minute period of stabilisation, the animals began a 20 minute baseline period, followed by one of the treatments described below. Parameters were recorded for 20 minutes before (T0) and 120 minutes after treatment, divided into six 20 minute periods (T1, T2, T3, T4, T5, T6). In each period, respiratory and haemodynamic data were continuously recorded, and renal data was recorded at the end of each period.

The following treatment groups were defined for this experiment:

- Group K, n = 6, received ketoprofen (Ketofen, 100 mg.mL^-1^, Merial, Lyon, France) (2 mg.kg^-1^ completed with saline; total volume of 0.04 mL.kg^-1^),
- Group KiNO, n = 6, received ketoprofen (2 mg.kg^-1^ completed with saline; total volume of 0.04 mL.kg^-1^) and 40 ppm iNO added to O_2_ throughout the experiment,
- Group C, n = 6, received a placebo-control injection (saline solution, 0.04 mL.kg^-1^) and inhaled 100% O_2_.

### Inhaled NO administration

A mixture of NO-N_2_ (Cylinder NO-N_2_ 1000 ppm, < 5 ppm NO_2_, Air Liquide Santé, Lyon, France) was administered cyclically into the inspiratory limb of the ventilator using a commercialised injector (Opti-NO^®^, Air Liquide Santé, Lyon, France). Based on our previous work [15], we chose to reproduce the renal effects of a dose of 40 ppm inhaled NO in a similar setting. The inspired NO and NO_2_ concentrations were monitored with an electrochemical analyzer (Polytron NO/NO_2_, Dräger A.G., Lübeck, Germany).

### Cardiovascular and respiratory monitoring

Systolic, mean and diastolic SAPs and PAPs, heart rate and electrocardiogram were continuously recorded (AcqKnowledge version 3.5.3^®^ for Windows 95/98, Biopac Systems Inc, Goleta, California, U.S.A.) and computed every 5 min. A non-invasive CO_2_ and anaesthetic gas monitor (NiCO^®^, Novametrix Medical Systems Inc, Wallingford, Connecticut, U.S.A.) was also used to calculate cardiac output (CO), based on the indirect Fick's principle with partial re-inhalation of CO_2_, and respiratory parameters: end-tidal CO_2_ concentration (ETCO_2_), respiratory rate and pulsed arterial haemoglobin saturation in oxygen (SpO_2_). These parameters were reported every 10 min. In Experiment I, CO values of group C calculated from the NiCO^®^ monitor were compared to those of the thermodilution method. During iNO administration, NiCO^®^ measurement of CO was not possible because interferences with the re-inhalation loop tended to facilitate NO_2_ formation and was replaced by thermodilution CO evaluation.

### Renal parameters measurement

Urinary output (UO), glomerular filtration rate (GFR) and RBF were assessed throughout the experiment.

Calculation of inulin clearance determined GFR. The initial bolus consisted in 2.6 g of inulin dissolved in 10.0 mL of sterile water for injection, 10.0 mL of phosphate buffered saline solution, and 0.9% sodium chloride solution (q.s.p. 50.0 mL); pH was adjusted to 7.4 with sodium hydroxide. Immediately after bolus administration, CRI was started at 1.0 mL.min^-1^ with a pump. CRI was prepared as follows: 4.0 g of inulin dissolved in 5.0 mL of sterile water for injection, 10.0 mL of phosphate buffered saline, and 0.9% sodium chloride solution (q.s.p. 200.0 mL), pH was adjusted to 7.4 with sodium hydroxide. Total infusion time was adjusted to the experiment time. Inulin concentration was measured in urine and plasma with an enzymatic assay [17]; GFR was considered equal to inulin clearance, and was calculated with the standard clearance formula:

$${\text{GFR}=\text{Clu}}_{\text{inulin}}=\text{UO x }\left({\text{Cu}}_{\text{inulin}}{\text{/Cp}}_{\text{inulin}}\right)$$

Where Clu_inulin_ and UO are the renal inulin clearance and the urine output (mL.min^-1^) respectively, and Cu_inulin_ and Cp_inulin_ are the inulin urine and arterial plasma concentration, respectively.

Right RBF measured by ultrasonic flowmetry (RBFul) was continuously registered. Data was treated with the AcqKnowledge^®^ acquisition software. In Experiment I, in order to evaluate the influence of the flow probe on renal function, RBF was also monitored with the PAH clearance method (RBF_PAH_): 2.0 g of PAH were added to the 50.0 mL inulin i.v. bolus and 0.8 g of PAH was added to the 200.0 mL inulin CRI solution. Both urine and plasma PAH concentrations were measured using a colorimetric method [18]. For estimation of RBF, renal plasma flow (RPF) was first assessed using the following formula:

$${\text{RPF}}_{\text{PAH}}{=\text{ Clu}}_{\text{PAH}}{\text{/E}}_{\text{PAH}}$$

where Clu_PAH_ represents renal clearance of PAH and E_PAH_ renal PAH extraction coefficient (considered as similar to the human value [19] and estimated at 0.9 [20] as previously validated in pigs by our group [15]).

Clu_PAH_ was calculated as followed:

$${\text{Clu}}_{\text{PAH}}=\text{ UO x }\left({\text{Cu}}_{\text{PAH}}{\text{/Cp}}_{\text{PAH}}\right)$$

Where Clu_PAH_ and UO are the renal PAH clearance and the urine output (mL.min^-1^) respectively, and Cu_PAH_ and Cp_PAH_ are the PAH urine and arterial plasma concentration, respectively.

Finally, RBF_PAH_ was calculated with the following equation:

$${\text{RBF}}_{\text{PAH}}{=\text{RPF}}_{\text{PAH}}\text{/}\left(\text{1-Ht}\right)$$

where Ht represents haematocrit.

To test the possible influence of the ultrasonic probe on the right renal function, we compared UO, GFR, and RBF_PAH_ in both kidneys, in addition to the total (right + left) values for these parameters.

### Biochemical analyses

Arterial blood was collected into 1.0 mL heparinised syringes for blood gas analysis (Omni 5^®^, AVL Medical Instruments, Cergy-Pontoise, France). Those included oxygen arterial tension (PaO_2_), carbon dioxide arterial tension (PaCO_2_), pH, bicarbonate (HCO_3_^-^) concentration, base excess (BE), electrolytes (sodium, potassium, chloride) and Ht. Arterial blood (5.0 mL) was collected in lithium-heparin tubes, centrifuged (3000 x *g* during 6 min), aliquoted and stored at -80°C pending analysis of total plasma protein (MASTER 3M refractometer^®^, Atago CO, Tokyo, Japan), osmolality (Osmomat 030^®^, Fisher Bioblock Scientific, Illkirch, France), as well as inulin/PAH dosages.

### Data collection

Continuous recording of systolic, mean and diastolic SAPs and PAPs, heart rate, CO, ETCO_2_, respiratory rate, and SpO_2_ was divided in 20 minute periods and averaged separately.

Thirty minutes after inulin/PAH CRI initiation, following steady state achievement, urine produced by each kidney was collected and discarded. Blood and urine were then collected for the determination of inulin/PAH concentration. Starting at time = T0–10 min, arterial blood was collected at 20 minute intervals on 3 (Experiment I) or 7 (Experiment II) occasions. Starting at time = T0, urine was collected individually from both ureters at 20 minute intervals on 3 (Experiment I) or 7 (Experiment II) occasions. Total UO was calculated at each interval. All samples were stored at -80°C pending analysis.

### Statistical analysis

Data was analysed with SAS 9.1 for Windows (SAS Institute Inc, Cary, North-Carolina, U.S.A.). To evaluate the stability of the model (or within-time variation), a linear mixed-effect model for repeated measures with maximum estimation was used to assess the influence of time on cardiovascular, respiratory and renal parameters in both Experimental phases. Exploratory data analysis was secondly performed to identify potential relationships between variables. Afterwards, a linear mixed-effect model for repeated measures with maximum estimation was used to assess determinants of the time-course of UO percent change with respect to baseline value. In this model, measurement time, treatment group, CO, RBF and GFR were included as fixed factors, and pig within treatment group was a random variable. In addition, a selected number of their double and triple interactions were included in the model, based on the results of the exploratory data analysis. The variance-covariance matrix of the data was modelled according to a strategy described by Littell *et al*. [21]. Briefly, a mixed-effect model containing no interaction was estimated with a free covariance structure, and the model was then re-estimated with more parsimonious covariance matrices (*e*.*g*., compound symmetry, first-order autoregressive, and Toeplitz), which structures resembled that of the unstructured covariance matrix. The final covariance model was selected according to the value of the Schwarz Bayesian Criterion [21]. Finally, least-square means of time*treatment were calculated to show the effect of treatment on the temporal trends of UO. A p-value < 0.05 was considered as significant, and allowed to reject null hypothesis. Estimates were shown as mean ± standard deviation, unless stated otherwise. Incomplete data or values apart from 95% confidence interval were excluded from statistical analyses.

## Results

### Experimental phase I: Reproduction of inhaled NO renal effects and methods validation

#### Cardiovascular parameters

In the group C, changes in heart rate were not significant (p = 0.46), and a small (-6.5±2.8%) but significant (p = 0.04) decrease in CO was observed during the REC period, while remaining within normal range values (82.8 to 139.3 mL.min-1 for the 6 pigs). Moreover, the determination coefficient between both CO measurement methods (thermodilution and NiCO) was excellent (r^2^ = 0.92). In the group iNO, no significant variation in heart rate or CO was found within time, and inter-groups comparisons revealed no significant difference at any period (p > 0.47). A significant increase in mean SAP was found at period REC, in both groups, iNO (+9±5.8%; p = 0.04) and C (+15.3±8.2%; p = 0.03), without significant difference between groups (P>0.63). Mean PAP decreased significantly (-20.1±7.1%) during NO inhalation (p = 0.008) while constant in group C (p = 0.69). No significant inter-group difference was observed at any time (p = 0.32) for mean PAP.

#### Renal parameters

**Fig 1:**
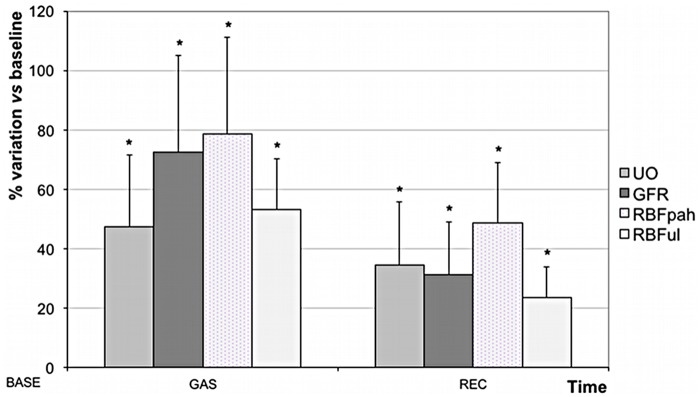
Variation of renal parameters during (GAS period) and after (REC period) inhalation of nitric oxide (NO). Data are expressed in percentage of variation in comparison with baseline value (mean±SD). UO = urinary output; GFR = glomerular filtration rate; RBF_UL_ = renal blood flow measured by ultrasonic flow probe; RBF_PAH_ = renal blood flow measured by PAH clearance method. * p < 0.05: significant intra-group variation in comparison with baseline value.

All renal parameters (UO, GFR, RBF_PAH_ and RBF_UL_) remained stable (p > 0.86) in group C. NO inhalation resulted in an important and significant increase in renal parameters (Fig 1): RBF_PAH_ (+78.6±37.6%; p = 0.004), RBF_UL_ (+53.2±17.2%; p = 0.008); GFR (+72.6±32.5%; p = 0.006) and UO (+47.4±24.2%; p = 0.01). During period REC, renal parameters decreased in the group iNO, but remained more elevated (+48.7±20.2%, +23.5±10.2%; +31.3±17.8%, and +34.6±21.2%, respectively) than baseline values (p < 0.01).

RBF_UL_ followed the same slope than RBF_PAH_ with a significant correlation between both RBF measurement methods (r^2^ = 0.83). Moreover, left and right kidney RBF_PAH_ values, in both groups, were not different and correlated.

#### Other parameters

Respiratory rate, arterial blood gases (PaCO_2_, HCO_3_^-^, BE), pH, ETCO_2_, SpO_2_, electrolytes, total plasma protein and osmolality did not vary significantly and stood within normal ranges. An increase in PaO_2_ was statistically supported (p = 0.02) from BASE (414.1±43.2 mmHg) to GAS (441.8±50.6).

### Phase II: Effect of ketoprofen associated to inhaled NO administration

#### Cardiovascular parameters

Heart rate, CO, mPAP and mSAP remained within normal ranges for all pigs throughout the experiment (Table 1). However, the CO decreased regularly with time in group C, whereas this decrease was short lasting in groups K and KiNO. Heart rate did not significantly vary in any group during the duration of the experiment (p > 0.37). For all groups, mean SAP increases significantly overtime (p < 0.0001), reaching a plateau value (+14.8–18.5%) by T3. Mean PAP remained stable in groups C and K (p > 0.2) whereas it decreased significantly from T1 to T6 for group KiNO (p < 0.001) with a maximal decrease of -24.7±11.3% at T4.

**Table 1:** Variations of haemodynamic and renal parameters over time in phase II.

Parameter	Time	T0 (Basal value)	T1 (% change)	T2 (% change)	T3 (% change)	T4 (% change)	T5 (% change)	T6 (% change)
**CO (mL/min)**	Group C	104.7±22.5	-2.8±5.3	-5.3±3.6	-9.4±5.0*	-10.3±3.7*	-12.9±4.4*	-12.5±7.2*
	Group K	106.1±20.8	-4.5±4.4	+0.1±2.6	-4.7±3.2	-6.2±3.8*	-5.3±5.9	-1.4±8.4
	Group KiNO	109.5±24.7	-0.7±2.6	-4.7±3.1	-7.8±3.1*	-8.9±3.3*	-3.6±4.5	-2.2±4.8
**mSAP (mmHg)**	Group C	82.6±9.71	+4.8±3.1	+9.6±7.5	+15.6±8.2*	+14.8±8.1*	+16.1±9.1*	+15.8±9.2*
	Group K	71.3±13.3	+9.1±5.7	+12.2±7.8	+18.2±8.9*	+17.1±9.3*	+15.3±9.7*	+15.8±8.8*
	Group KiNO	86.6±6.1	+7.7±3.7	+14.4±6.5	+14.8±7.3*	+16.5±7.6*	+15.7±8.7*	+14.7±9.8
**RBF**_**UL**_ **(mL/min)**	Group C	6.7±2.0	-0.7±3.2	-6.5±5.9	-6.7±6.9	-5.8±5.6	-5.6±4.8	-3.5±5.8
	Group K	5.3±0.8	-17.5±4.1*	-19.0±7.1*	-16.9±7.8*	-17.1±8.1*	-18.11±9.6*	-14.7±7.1*
	Group KiNO	5.2±1.5	-7.2±4.9	-8.2±5.3	-6.4±4.9	-6.9±5.4	-5.6±5.8	-3.5±4.6
**GFR (mL/min)**	Group	1.9±0.6	-5.1	-2.6	-2.8	+0.6	+9.7	+5.1
	C		±11.5	±8.6	±9.9	±13.1	±19.6	±14.8
	Group K	1.7±0.5	-19.5±9.1*	-26.6±10.4*	-25.3±7.1*	-21.1±9.8*	-20.9±8.9*	-19.3±11.2
	Group KiNO	1.5±0.8	-11.1±12.7	+0.0±13.4	-1.9±16.1	-7.1±17.9	-3.5±18.6	-2.8±17.7
**UO (μL/min)**	Group C	24.2±11.5	+0.9±5.5	+1.9±11.3	-0.2±13.2	+7.3±12.4	+17.4±13.9	+8.9±16.1
	Group K	20.9±4.1	-24.2±7.8*	-26.8±8.4*	-30.3±10.5*	-26.9±9.6*	-25.1±9.4*	-22.1±7.1*
	Group KiNO	23.4±8.5	-6.7±8.1	-10.8±12.1	+3.7±12.2	+3.5±13.1	-1.8±14.0	-2.7±10.7

At the end of the baseline period (T0), piglets were randomly allocated to receive an IV injection of sterile placebo-control and inhaled 100% O2 (n = 6; Group C), an IV injection of ketoprofen and inhaled 100% O2 (n = 6; Group K), or IV ketoprofen and 40 ppm inhaled NO (n = 6; Group KiNO). Cardiac output (CO), mean systemic arterial blood pressure (mSAP), renal blood flow monitored with an ultrasonic probe (RBF_UL_), glomerular filtration rate (GFR) and urinary output (UO) of anaesthetised pigs were followed-up for seven successive 20-min periods (T0 to T6), with treatments administered at T1. Data are presented as mean percentage of change (±SD) from T1 to T6, compared with the mean value recorded in period T0.

* indicates a significant difference within time-points for the same parameter compared to baseline evaluated with a fixed-model for repeated measures.

#### Renal parameters

For groups C and KiNO, no significant temporal variation was noted regarding GFR (p > 0.6) and UF (p > 0.44). The decrease observed in RBF_UL_ was not significant (p > 0.09), but presented a nadir of -6.7±6.9% at T3 in group C, and -8.2±5.3% at T2 in group KiNO, partially mimicking the time course of CO (r^2^ = 0.44) with a progressive return to baseline values. The evolution of renal parameters was different in group K, with a significant and constant decrease observed for RBF_UL_, GFR and UO (p < 0.02) (Table 1).

**Fig 2:**
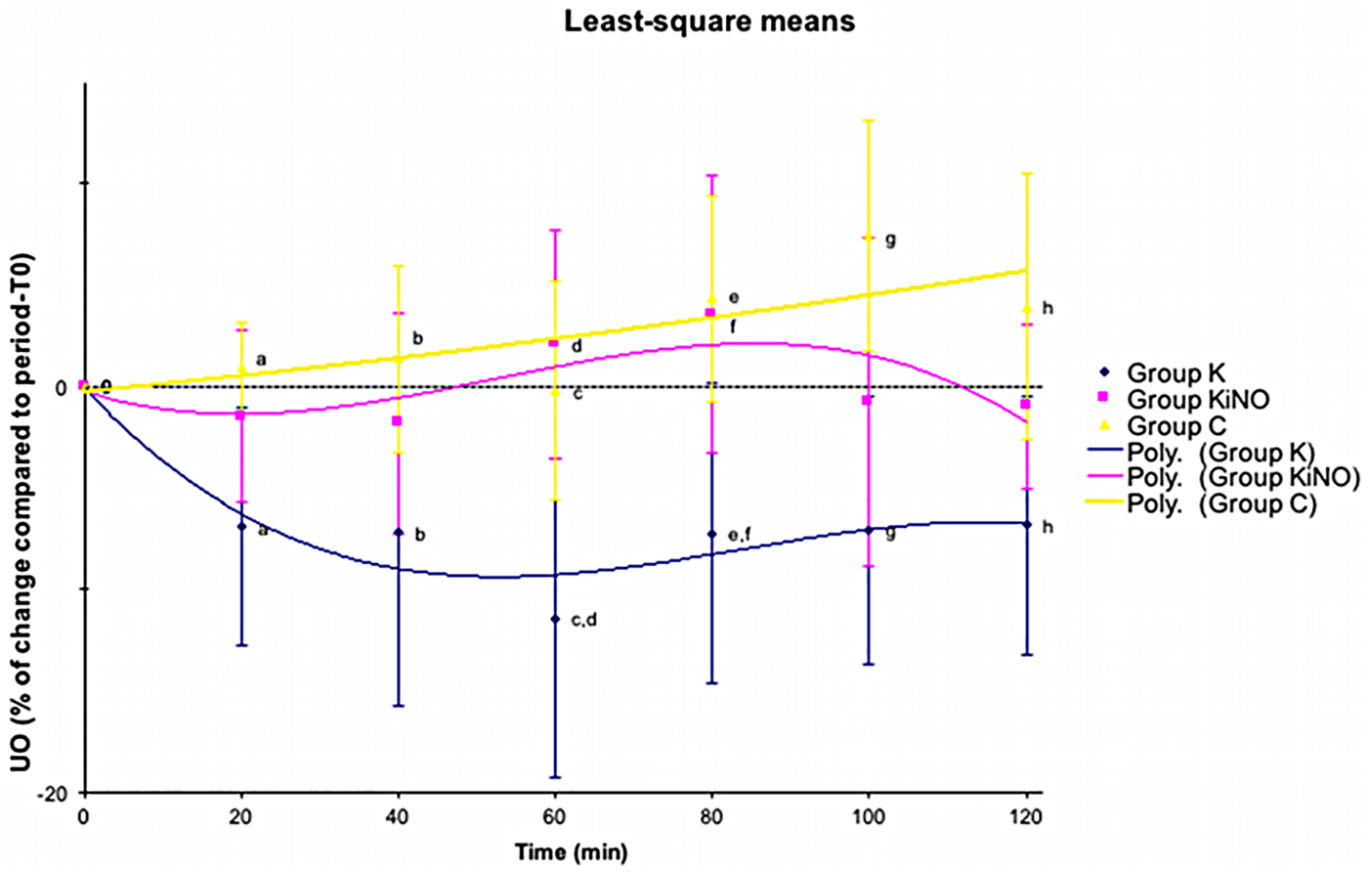
Least-square means of time*treatment differences within group (intra-group variation) and between groups (inter-group variation) of urinary output (UO) with measurement time, treatment group, cardiac output, renal blood flow, and glomerular filtration rate as fixed factors. Data are presented as mean percentage of change (±SE) compared with the mean value recorded in period-T0 as well as with the 2nd-order polynomial trend curve for each group. Similar super-script letters mean statistical significant difference between time-points of different groups.

Based on exploratory analysis, UO was selected as the most significant parameter to be analysed for assessment of renal function with CO, RBF_UL_, GFR as fixed factors. For UO least-square means of time*treatment intra-group variation, Fig 2 clearly demonstrates the absence of change within time for UO in groups C (p = 0.86) and KiNO (p = 0.86). A significant difference was observed for group K between time points T1 and T6 compared to period-T0 (p = 0.03).

Moreover, inter-group comparison (Fig 2) showed that time points T1 to T6 in group K were significantly lower than similar time points in group C. Time points T3 and T4 in group K were significantly lower than similar time points in group KiNO. No inter-group difference was found between group KiNO and group C at any time point. No difference was found when comparing right and left kidneys UO and GFR in any group.

#### Other parameters

Respiratory rate, ETCO_2_ and blood gases analyses (PaO_2_, PaCO_2_) were stable throughout the experiment for every group (p > 0.25), without difference between groups (p > 0.2 for all four parameters). Total plasma protein, osmolality, arterial blood electrolytes and acid-base status (pH, HCO_3_^-^ and BE) remained stable and within normal range for all groups.

## Discussion

The objectives of this study were to confirm the renal effects of inhaled NO in pseudo-normovolaemic anaesthetised piglets and to evaluate whether iNO could compensate or prevent the decrease in RBF induced by ketoprofen, a conventional NSAID in the present condition. The main results of this study are a significant improvement of renal parameters (RBF, GFR, UO) following inhalation of NO, along with a moderate but significant variation in CO and SAP. A significant decrease in RBF, GFR and UO was observed following ketoprofen administered alone, while these parameters did not vary significantly when ketoprofen was given with iNO.

The experimental model aimed to obtain a reproducible alteration of renal haemodynamic that could mimic a clinical condition. To achieve this objective, a porcine model was chosen due to the functional and anatomical similarities with human kidney [22]. Moreover, a pseudo-normovolaemic condition was used to place the kidney in a vasodilatory PGs-dependant state and potentiate renal side effects of NSAIDs. Pseudo-normovolaemic condition is defined as a slight negative water balance induced in healthy anaesthetised animals by receiving minimal or no fluid therapy. This condition aimed to place the kidney in a vasodilatory PGs-dependent state and was obtained in our experimental setting by giving an infusion rate of fluids of 3.5 mL.kg^-1^·h^-1^ that is inferior to the conventionally recommended rate of 10 mL.kg^-1^.h^-1^ during anaesthesia [23]. This situation of altered volaemia, in addition of the cardiovascular effects of anaesthetic drugs, sustains the slight decrease in CO observed for groups C and K. However, this small decrease in CO is likely to be clinically insignificant.

Several parameters were measured to have a global estimation of renal function: UO, GFR, RBF. GFR is considered as the best overall index to estimate renal function [24]. It was assessed in our experiment by inulin clearance, the gold standard method, which appeared reliable in our experiments, as the recorded values were similar to previous reports [15]. Regarding RBF, PAH clearance, the reference method [25], was used in the preliminary Experiment I to validate the ultrasonic method and evaluate the potential effect of the flow probe on renal function. Both methods of RBF measurement were linearly correlated. Because no significant difference was found between right and left kidney values for UO, GFR and RBF_PAH_, we can conclude that the ultrasonic probe placed around the right renal artery did not have any major influence on RBF. Moreover, we obtained similar tendencies with data obtained by both methods, even though RBF_PAH_ values appeared higher. This inconvenient has already been described as a limitation of RBF estimation using a colorimetric PAH dosage method [26]. Our data are in accordance with data reported by Möckel *et al*. [27], who concluded that the ultrasonic method is a reliable technique to estimate RBF in pigs. Based on these data, this last method was used to assess RBF in Phase II. Although it is an invasive procedure, the ultrasonic method has the advantage to allow a continuous measurement of RBF and an early detection of variations.

NSAIDs-induced AKI is a well-documented complication, with both haemodynamic impairment and inflammation playing a role, but in an anaesthetic context, decreased renal perfusion appears as the principal cause [28]. In this experimental setting, ketoprofen, a conventional NSAID, induced a significant alteration in renal parameters, confirming the results of a previous study using a similar model [23]. In situations of compromised renal haemodynamics (such as hypovolemia or dehydration), vasodilatory PGs, primarily PGE2 and PGI2, are synthesised by COX enzymes to maintain an acceptable RBF and GFR, thwarting the effects of vasoconstricting substances (angiotensin II, vasopressin, *etc*.) involved in the autoregulation process [29]. By inhibiting non-selectively COX-1 and COX-2 enzymes in condition of altered volaemia, ketoprofen has participated to the impairment of kidney haemodynamics. The significant decrease in GFR and RBF in group K despite the maintenance of CO and SAP is in favour of an increased afferent arteriolar resistance.

COX-2 specific inhibitors have been proposed as a potential way of improving renal tolerance of anti-inflammatory drugs. A recent meta-analysis has reported the respective risk of different molecules in human patients: traditional NSAIDs were associated with greater risk of inducing AKI than selective COX-2 inhibitors [30]. However, for patients with low circulating volume, regulation of renal function appears dependent on PGs synthesised through the COX-2 pathway [31]. Thus, COX-2 inhibitors do not guaranty a good renal tolerance of anti-inflammatory treatment for the critical patients.

Based on the previous elements, the co-administration of a molecule presenting renal vasodilatory properties could constitute a potential way of improving the safety of NSAIDs on kidney haemodynamics. Among the drugs with local vasodilatory properties, NO, given in its inhaled form, has a special interest. In the kidney, it has numerous important functions, including renal haemodynamics regulation, maintenance of medullary perfusion, mediation of pressure-natriuresis, blunting of tubuloglomerular feedback, inhibition of tubular sodium reabsorption and modulation of renal sympathetic neural activity. The net effect of NO in the kidney is the promotion of natriuresis and diuresis [32]. Regarding its inhaled form, it is a well-established treatment for pulmonary hypertension: by inducing pulmonary vasodilatation in ventilated regions, it decreases PAP, attenuates intra-pulmonary shunting and optimizes ventilation-perfusion matching, and therefore oxygenation [33]. Previous studies and clinical trials claimed that iNO therapeutic effect was restricted to the pulmonary area due to its rapid degradation in the blood stream. However, several clinical and experimental reports have confirmed that iNO has also extra-pulmonary effects, some of them located in the kidney [15,34]. Our original data stating the renal effects of iNO [15] were clearly reproduced in Phase I. Even if the experimental conditions were not exactly similar (different anaesthesia and fluid therapy protocols), the magnitude of increase in RBF_PAH_ (+78.6% *vs*. +109%), GFR (+72.6% *vs*. +72.5%) and UO (+47.4% *vs*. +68.7%) was identical to this previous report [15]. In both experiments, iNO produced vasodilation of apparently both afferent and efferent arterioles, as indicated by the significant concomitant raise of GFR and RBF. Inhaled NO also induced a significant increase in UO, which confirms a common observation among human patients receiving iNO [35]. The results of the phase I experiments highlight that exogenous NO delivered at the kidney level can mimic renal physiological functions of endogenous NO. The mechanisms involved in the release of NO at distal sites remain to be determined, but several candidates have been proposed to explain extrapulmonary effect of iNO. Oxidation of NO to nitrate and/or nitrite, formation of S-nitrosothiols could thus represent potential transport forms of NO following its inhalation [15,34,36,37]. Cannon *et al*. [34] have also suggested that iNO could supply a deficiency of NO production in case of endothelium dysfunction. The short delay of action and persistence of renal effects in our experiment during period REC, after iNO administration was stopped, is in accordance with our hypothesis of NO-carriage by blood-borne molecules.

Despite their well-known existence, extra-pulmonary effects of iNO have seldom been evaluated as a therapeutic target. This application has been proposed against inflammation in diseases associated with a decrease in NO production [38], against ischaemia / reperfusion injuries [39–41]. Recently, it has been suggested that iNO might have a protective action on kidney function in an experimental model of endotoxinic shock, when combined with intravenous hydrocortisone [42]. In this last study, iNO was given at 30 ppm and renal function mainly assessed by UO, but the authors failed to find a significant effect and reported only a tendency to protection. Our experimental setting was based on the evaluation of several renal parameters, which allowed a more precise estimation of kidney function. In the Phase II experiment of our study, iNO administration concomitant to ketoprofen administration completely counteracted the NSAIDs-induced renal alterations. This confirms the interactions between nitrergic and prostanoid pathways [43] in the maintenance of renal function [44]. Arachidonic acid derived-products have been involved in the renal response to NO inhibition [45], but this is the first report of a compensatory effect of exogenous NO. Released from its transport forms after its inhalation, NO is also able to limit the renal vasoconstriction induced by prostanoids synthesis inhibition.

Although the results of this experiment are promising, we acknowledge some limitations. The intravenous anaesthetic protocol used in this experiment is not routinely used in human medicine but it aims to reproduce the experimental conditions reported in previous studies [15,23]. This protocol was also chosen to obtain a stabilised cardiovascular function and limit the peripheral vasodilation and hypotension associated with more commonly used intravenous and volatile anaesthetics. Interestingly, renoprotective effects have been reported with both propofol and inhalant agents, presumably due to a protective action against pro-inflammatory mediators [46,47]. Our study was not designed to evaluate the inflammatory cause of AKI, but mainly its haemodynamic origin. Complementary studies would thus be needed to assess the influence of these anaesthetic agents on renal haemodynamics in similar conditions. The dose and duration of administration of iNO would need to be further clarified, as we did not evaluate neither a dose-effect relationship, nor the evolution of renal function at the end of NO inhalation. As previously mentioned, AKI is not only mediated by haemodynamic impairment, nephritis may also play a role. NO given by inhalation may have anti-inflammatory properties [38] and may attenuate reperfusion injuries [40], but our experiment was not designed to assess this factor. Administration of iNO in some critical conditions remains questionable, as it has been associated with AKI in patients presenting an acute respiratory distress syndrome, but the cause of this complication is uncertain and remains to be elucidated [48]. Finally, the positive effect of iNO on kidney haemodynamics reported in our experimental conditions may be difficult to apply in a clinical setting due to the limited access and cost of iNO. However, these interesting results could be relevant regarding NO-donor compounds. These pharmacological entities might show similar effects by releasing NO through its transport forms, but this remains to be confirmed.

## Conclusion

The present study confirms the potential therapeutic benefit of the extrapulmonary effects of iNO. Some questions remain to be solved before a clinical application in human patients, such as the optimal dosage of iNO and its duration of administration. However, these results are promising for improvement of the renal tolerance of NSAIDs in the anaesthetised or critical patient. Further studies must be carried out to evaluate if other NO-donor molecules could mimic the results reported here.
